# Ethyl 2-methyl-6-(propan-2-yl­amino)-4-sulfanyl­idene-3*H*,11*H*-pyrimido[1,6-*c*]quinazoline-1-carboxyl­ate

**DOI:** 10.1107/S1600536812019952

**Published:** 2012-05-19

**Authors:** Hong-Xia Li, Yu-Su Song, Yong-nian Qu, Jiang-Bing Lu, Hong-Mei Wang

**Affiliations:** a College of Science, Naval University of Engineering, Wuhan 430033, People’s Republic of China; b Institute of Medicinal Chemistry, Hubei University of Medicine, Shi Yan 442000, People’s Republic of China

## Abstract

The title compound, C_18_H_22_N_4_O_2_S, contains a substituted pyrimidine ring fused to both a benzene ring and a substituted thioxopyrimidine ring. The pyrimidine and thioxopyrimidine rings adopt distorted chair conformations. In the crystal, adjacent mol­ecules are linked by pairs of N—H⋯S and N—H⋯O hydrogen bonds to generate centrosymmetric *R*_2_^2^(8) and *R*_2_^2^(16) loops, respectively. This combination leads to [100] chains of mol­ecules.

## Related literature

For further synthetic details, see: Li *et al.* (2007 ▶, 2008 ▶); Huang *et al.* (2009 ▶); Zeng *et al.* (2010 ▶). For a related structure, see: Li *et al.* (2010 ▶). For ring conformations, see: Cremer & Pople (1975 ▶).
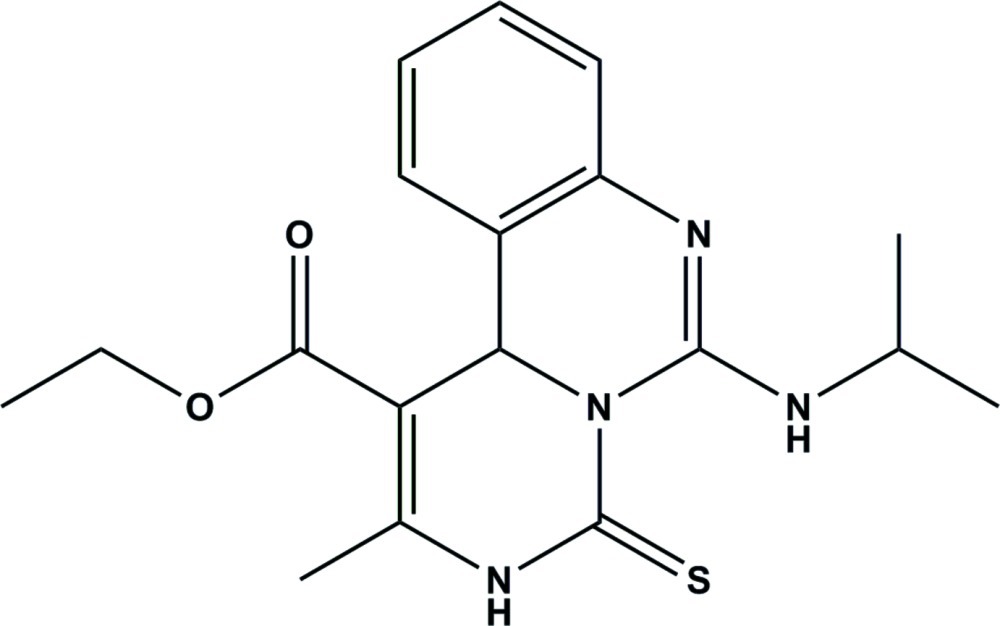


## Experimental

### 

#### Crystal data

C_18_H_22_N_4_O_2_S
*M* *_r_* = 358.46
Monoclinic, 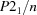

*a* = 9.4128 (3) Å
*b* = 10.5636 (5) Å
*c* = 19.2052 (6) Å
β = 102.347 (1)°
*V* = 1865.46 (12) Å^3^
*Z* = 4
Mo *K*α radiation
μ = 0.19 mm^−1^
*T* = 298 K
0.30 × 0.20 × 0.20 mm

#### Data collection

Bruker SMART CCD diffractometer
Absorption correction: multi-scan (*SADABS*; Sheldrick, 1996 ▶) *T*_min_ = 0.955, *T*_max_ = 0.962
12093 measured reflections
3857 independent reflections
3014 reflections with *I* > 2σ(*I*)
*R*_int_ = 0.076

#### Refinement

*R*[*F* ^2^ > 2σ(*F* ^2^)] = 0.050
*wR*(*F* ^2^) = 0.139
*S* = 1.03
3857 reflections
236 parameters
2 restraints
H atoms treated by a mixture of independent and constrained refinement
Δρ_max_ = 0.34 e Å^−3^
Δρ_min_ = −0.27 e Å^−3^

### 

Data collection: *SMART* (Bruker, 2001 ▶); cell refinement: *SAINT* (Bruker, 2001 ▶); data reduction: *SAINT*; program(s) used to solve structure: *SHELXS97* (Sheldrick, 2008 ▶); program(s) used to refine structure: *SHELXL97* (Sheldrick, 2008 ▶); molecular graphics: *PLATON* (Spek, 2009 ▶); software used to prepare material for publication: *SHELXTL* (Sheldrick, 2008 ▶).

## Supplementary Material

Crystal structure: contains datablock(s) global, I. DOI: 10.1107/S1600536812019952/hb6711sup1.cif

Structure factors: contains datablock(s) I. DOI: 10.1107/S1600536812019952/hb6711Isup2.hkl

Supplementary material file. DOI: 10.1107/S1600536812019952/hb6711Isup3.cml

Additional supplementary materials:  crystallographic information; 3D view; checkCIF report

## Figures and Tables

**Table 1:** Hydrogen-bond geometry (Å, °)

*D*—H⋯*A*	*D*—H	H⋯*A*	*D*⋯*A*	*D*—H⋯*A*
N2—H2*A*⋯O2^i^	0.86 (1)	2.26 (1)	3.102 (2)	165 (2)
N4—H4*A*⋯S1^ii^	0.87 (1)	2.42 (1)	3.2804 (15)	172 (2)

Symmetry codes: (i) 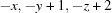; (ii) 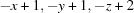.

**Fig. 1.**
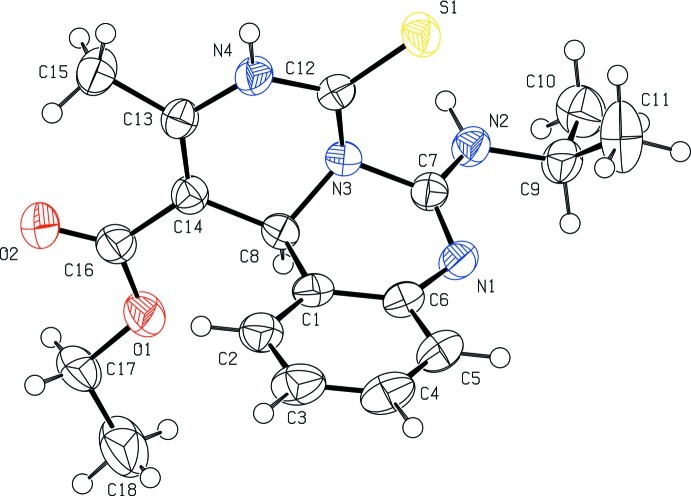
View of the molecule showing displacement ellipsoids drawn at the 50% probability level.

## supplementary crystallographic information

### Comment

In recent years, we have been engaged in the preparation of heterocyclic derivatives using the aza-Wittig reaction (Li *et al.* 2007, 2008; Huang *et al.* 2009; Li *et al.* 2010; Zeng *et al.*, 2010). We present here the crystal structure of the title compound (Fig. 1). The molecule contains a substituted pyrimidine ring fused to a benzene ring and a substituted thioxopyrimidine ring. The centre ring of pyrimidine moiety adopts a distored chair conformation [φ = 208.8 (2)° and θ = 68.85 (19)°, Puckering Amplitude = 0.5502 (17)Å], and the substituted thioxopyrimidine ring also show a distorted chair form [φ = 214.6 (4)° and θ = 113.9 (3)°, Puckering Amplitude = 0.3827 (17)Å] (Cremer & Pople, 1975). In the crystal, there are N—H···O and N—H···S (Table 1) hydrogen bonds.

### Experimental

To a solution of iminophosphorane prepared according to Li *et al.* (2010) (0.55 g, 1 mmol) in CH_3_CN (10 mL) was added phenylisocyanate (0.12 g, 1 mmol) under nitrogen at room temperature. After stirred for 2 h at room temperature, K_2_CO_3_ (0.014 g,0.1 mmol) was added and the mixture was stirred for 1 h. The solvent was removed off under reduced pressure and the residue was recrystallized from methylene dichloride and ethanol to give the title compound (I) in yield of 85% (m.p. 490 K). Colourless blocks were obtained from a dichloromethane solution at room temperature.

### Refinement

The H atoms attached to atoms N2 and N4 was located in a difference Fourier map and allowed to ride on their parent atom with a restraint of N—H = 0.86 Å. Other H atoms were placed at calculated positions and treated as riding atoms, with C—H = 0.96–0.97 Å, and *U*_iso_(H) = 1.2 *U*_eq_(C) or 1.5 *U*_eq_(C) for methyl H atoms.

### Crystal data

**Table:**

C_18_H_22_N_4_O_2_S	*F*(000) = 760.0
*M**_r_* = 358.46	*D*_x_ = 1.288 Mg m^−^^3^
Monoclinic, *P*2_1_/*n*	Mo *K*α radiation, λ = 0.71073 Å
Hall symbol: -P 2yn	Cell parameters from 4329 reflections
*a* = 9.4128 (3) Å	θ = 2.2–27.7°
*b* = 10.5636 (5) Å	µ = 0.19 mm^−^^1^
*c* = 19.2052 (6) Å	*T* = 298 K
β = 102.347 (1)°	Block, colorless
*V* = 1865.46 (12) Å^3^	0.30 × 0.20 × 0.20 mm
*Z* = 4	

### Data collection

**Table:**

Bruker SMART CCD diffractometer	3857 independent reflections
Radiation source: fine-focus sealed tube	3014 reflections with *I* > 2σ(*I*)
Graphite monochromator	*R*_int_ = 0.076
φ and ω scans	θ_max_ = 26.5°, θ_min_ = 2.2°
Absorption correction: multi-scan (*SADABS*; Sheldrick, 1996)	*h* = −11→11
*T*_min_ = 0.955, *T*_max_ = 0.962	*k* = −13→10
12093 measured reflections	*l* = −24→24

### Refinement

**Table:**

Refinement on *F*^2^	Primary atom site location: structure-invariant direct methods
Least-squares matrix: full	Secondary atom site location: difference Fourier map
*R*[*F*^2^ > 2σ(*F*^2^)] = 0.050	Hydrogen site location: inferred from neighbouring sites
*wR*(*F*^2^) = 0.139	H atoms treated by a mixture of independent and constrained refinement
*S* = 1.03	*w* = 1/[σ^2^(*F*_o_^2^) + (0.0772*P*)^2^ + 0.1069*P*] where *P* = (*F*_o_^2^ + 2*F*_c_^2^)/3
3857 reflections	(Δ/σ)_max_ = 0.025
236 parameters	Δρ_max_ = 0.34 e Å^−^^3^
2 restraints	Δρ_min_ = −0.27 e Å^−^^3^

### Special details

**Table:**

Geometry. All esds (except the esd in the dihedral angle between two l.s. planes) are estimated using the full covariance matrix. The cell esds are taken into account individually in the estimation of esds in distances, angles and torsion angles; correlations between esds in cell parameters are only used when they are defined by crystal symmetry. An approximate (isotropic) treatment of cell esds is used for estimating esds involving l.s. planes.
Refinement. Refinement of F^2^ against ALL reflections. The weighted R-factor wR and goodness of fit S are based on F^2^, conventional R-factors R are based on F, with F set to zero for negative F^2^. The threshold expression of F^2^ > 2sigma(F^2^) is used only for calculating R-factors(gt) etc. and is not relevant to the choice of reflections for refinement. R-factors based on F^2^ are statistically about twice as large as those based on F, and R- factors based on ALL data will be even larger.

### Fractional atomic coordinates and isotropic or equivalent isotropic displacement parameters (Å^2^)

**Table:**

	*x*	*y*	*z*	*U*_iso_*/*U*_eq_	
C1	0.09011 (18)	0.72990 (17)	0.84262 (9)	0.0364 (4)	
C2	0.0719 (2)	0.67844 (18)	0.77505 (10)	0.0462 (5)	
H2	0.0294	0.5991	0.7656	0.055*	
C3	0.1170 (3)	0.7450 (2)	0.72128 (10)	0.0570 (6)	
H3	0.1042	0.7107	0.6758	0.068*	
C4	0.1809 (3)	0.8623 (2)	0.73568 (11)	0.0558 (6)	
H4	0.2120	0.9067	0.6998	0.067*	
C5	0.1991 (2)	0.91431 (19)	0.80297 (10)	0.0494 (5)	
H5	0.2422	0.9935	0.8120	0.059*	
C6	0.1535 (2)	0.84940 (17)	0.85729 (9)	0.0380 (4)	
C7	0.17531 (19)	0.83292 (16)	0.97816 (9)	0.0348 (4)	
C8	0.04635 (19)	0.66650 (16)	0.90571 (9)	0.0345 (4)	
H8	−0.0437	0.7061	0.9126	0.041*	
C9	0.2171 (2)	1.00033 (18)	1.06939 (10)	0.0466 (5)	
H9	0.1470	1.0559	1.0387	0.056*	
C10	0.1933 (3)	1.0103 (2)	1.14433 (12)	0.0612 (6)	
H10A	0.0963	0.9833	1.1453	0.092*	
H10B	0.2063	1.0965	1.1601	0.092*	
H10C	0.2621	0.9573	1.1753	0.092*	
C11	0.3679 (3)	1.0396 (2)	1.06328 (15)	0.0795 (8)	
H11A	0.4385	0.9897	1.0953	0.119*	
H11B	0.3823	1.1275	1.0754	0.119*	
H11C	0.3791	1.0265	1.0153	0.119*	
C12	0.28831 (19)	0.62791 (16)	0.97962 (9)	0.0343 (4)	
C13	0.13802 (19)	0.44969 (16)	0.92654 (9)	0.0363 (4)	
C14	0.02360 (19)	0.52521 (16)	0.90196 (9)	0.0355 (4)	
C15	0.1435 (2)	0.30767 (17)	0.92606 (12)	0.0502 (5)	
H15A	0.1103	0.2753	0.9665	0.075*	
H15B	0.2417	0.2804	0.9284	0.075*	
H15C	0.0821	0.2765	0.8830	0.075*	
C16	−0.1233 (2)	0.47530 (18)	0.87161 (9)	0.0404 (4)	
C17	−0.3692 (2)	0.5353 (2)	0.82596 (12)	0.0552 (5)	
H17A	−0.4100	0.4905	0.8612	0.066*	
H17B	−0.3745	0.4806	0.7849	0.066*	
C18	−0.4504 (3)	0.6539 (3)	0.8048 (2)	0.0979 (11)	
H18A	−0.4431	0.7077	0.8457	0.147*	
H18B	−0.5508	0.6345	0.7856	0.147*	
H18C	−0.4101	0.6966	0.7693	0.147*	
S1	0.44958 (5)	0.68615 (5)	1.01900 (3)	0.04898 (19)	
N1	0.16807 (17)	0.90727 (14)	0.92474 (8)	0.0399 (4)	
N2	0.18611 (17)	0.86974 (14)	1.04561 (8)	0.0387 (4)	
H2A	0.191 (2)	0.8115 (14)	1.0774 (8)	0.046*	
N3	0.16359 (15)	0.69732 (13)	0.96840 (7)	0.0326 (3)	
N4	0.27056 (16)	0.50620 (14)	0.95704 (8)	0.0393 (4)	
H4A	0.3503 (15)	0.4619 (16)	0.9646 (10)	0.047*	
O1	−0.21898 (14)	0.57011 (13)	0.85569 (8)	0.0525 (4)	
O2	−0.15658 (16)	0.36556 (14)	0.86187 (8)	0.0569 (4)	

### Atomic displacement parameters (Å^2^)

**Table:**

	*U*^11^	*U*^22^	*U*^33^	*U*^12^	*U*^13^	*U*^23^
C1	0.0333 (9)	0.0385 (10)	0.0363 (9)	0.0059 (8)	0.0045 (7)	0.0066 (7)
C2	0.0516 (12)	0.0455 (11)	0.0392 (10)	0.0030 (9)	0.0045 (9)	0.0016 (8)
C3	0.0744 (15)	0.0612 (14)	0.0352 (10)	0.0142 (12)	0.0113 (10)	0.0055 (9)
C4	0.0695 (15)	0.0559 (13)	0.0460 (11)	0.0109 (11)	0.0215 (10)	0.0180 (10)
C5	0.0567 (13)	0.0425 (11)	0.0509 (11)	0.0035 (9)	0.0163 (10)	0.0131 (9)
C6	0.0387 (10)	0.0348 (10)	0.0401 (9)	0.0068 (8)	0.0077 (8)	0.0051 (7)
C7	0.0306 (9)	0.0329 (9)	0.0409 (9)	0.0019 (7)	0.0077 (7)	−0.0004 (7)
C8	0.0298 (8)	0.0366 (10)	0.0357 (8)	0.0006 (7)	0.0041 (7)	0.0004 (7)
C9	0.0554 (12)	0.0361 (10)	0.0481 (11)	0.0040 (9)	0.0107 (9)	−0.0051 (8)
C10	0.0690 (15)	0.0603 (14)	0.0544 (12)	0.0072 (12)	0.0140 (11)	−0.0168 (10)
C11	0.0865 (19)	0.0674 (17)	0.0933 (19)	−0.0312 (14)	0.0387 (16)	−0.0277 (14)
C12	0.0369 (9)	0.0324 (9)	0.0338 (8)	0.0020 (7)	0.0077 (7)	0.0034 (7)
C13	0.0379 (10)	0.0346 (9)	0.0379 (9)	−0.0025 (7)	0.0113 (7)	0.0015 (7)
C14	0.0362 (9)	0.0358 (10)	0.0348 (9)	−0.0028 (7)	0.0085 (7)	0.0006 (7)
C15	0.0483 (12)	0.0375 (11)	0.0662 (13)	−0.0022 (9)	0.0150 (10)	0.0009 (9)
C16	0.0414 (10)	0.0440 (11)	0.0356 (9)	−0.0047 (9)	0.0074 (8)	0.0039 (8)
C17	0.0373 (11)	0.0642 (14)	0.0587 (12)	−0.0093 (10)	−0.0021 (9)	−0.0010 (10)
C18	0.0541 (16)	0.0719 (18)	0.148 (3)	0.0067 (13)	−0.0231 (17)	−0.0301 (18)
S1	0.0332 (3)	0.0401 (3)	0.0684 (4)	0.00183 (19)	−0.0007 (2)	−0.0082 (2)
N1	0.0450 (9)	0.0325 (8)	0.0420 (8)	0.0024 (7)	0.0088 (7)	0.0039 (6)
N2	0.0451 (9)	0.0327 (8)	0.0389 (8)	0.0017 (7)	0.0104 (7)	−0.0004 (6)
N3	0.0310 (7)	0.0323 (8)	0.0335 (7)	0.0012 (6)	0.0043 (6)	0.0010 (6)
N4	0.0338 (8)	0.0320 (8)	0.0507 (9)	0.0033 (6)	0.0057 (7)	0.0006 (7)
O1	0.0345 (7)	0.0504 (9)	0.0675 (9)	−0.0043 (6)	−0.0002 (6)	−0.0017 (7)
O2	0.0522 (9)	0.0447 (9)	0.0673 (9)	−0.0126 (7)	−0.0016 (7)	0.0034 (7)

### Geometric parameters (Å, º)

**Table:**

C1—C2	1.384 (2)	C11—H11A	0.9600
C1—C6	1.399 (3)	C11—H11B	0.9600
C1—C8	1.516 (2)	C11—H11C	0.9600
C2—C3	1.389 (3)	C12—N4	1.356 (2)
C2—H2	0.9300	C12—N3	1.362 (2)
C3—C4	1.380 (3)	C12—S1	1.6625 (18)
C3—H3	0.9300	C13—C14	1.342 (2)
C4—C5	1.381 (3)	C13—N4	1.394 (2)
C4—H4	0.9300	C13—C15	1.501 (2)
C5—C6	1.390 (3)	C14—C16	1.478 (2)
C5—H5	0.9300	C15—H15A	0.9600
C6—N1	1.412 (2)	C15—H15B	0.9600
C7—N1	1.282 (2)	C15—H15C	0.9600
C7—N2	1.335 (2)	C16—O2	1.205 (2)
C7—N3	1.446 (2)	C16—O1	1.338 (2)
C8—N3	1.485 (2)	C17—O1	1.454 (2)
C8—C14	1.507 (2)	C17—C18	1.479 (3)
C8—H8	0.9800	C17—H17A	0.9700
C9—N2	1.463 (2)	C17—H17B	0.9700
C9—C10	1.507 (3)	C18—H18A	0.9600
C9—C11	1.507 (3)	C18—H18B	0.9600
C9—H9	0.9800	C18—H18C	0.9600
C10—H10A	0.9600	N2—H2A	0.861 (9)
C10—H10B	0.9600	N4—H4A	0.870 (9)
C10—H10C	0.9600		
			
C2—C1—C6	120.35 (16)	H11A—C11—H11C	109.5
C2—C1—C8	125.15 (16)	H11B—C11—H11C	109.5
C6—C1—C8	114.50 (15)	N4—C12—N3	114.64 (15)
C1—C2—C3	120.16 (19)	N4—C12—S1	122.31 (13)
C1—C2—H2	119.9	N3—C12—S1	123.04 (13)
C3—C2—H2	119.9	C14—C13—N4	118.16 (16)
C4—C3—C2	119.69 (19)	C14—C13—C15	128.17 (17)
C4—C3—H3	120.2	N4—C13—C15	113.67 (16)
C2—C3—H3	120.2	C13—C14—C16	122.63 (17)
C3—C4—C5	120.42 (19)	C13—C14—C8	118.46 (16)
C3—C4—H4	119.8	C16—C14—C8	118.91 (15)
C5—C4—H4	119.8	C13—C15—H15A	109.5
C4—C5—C6	120.66 (19)	C13—C15—H15B	109.5
C4—C5—H5	119.7	H15A—C15—H15B	109.5
C6—C5—H5	119.7	C13—C15—H15C	109.5
C5—C6—C1	118.72 (17)	H15A—C15—H15C	109.5
C5—C6—N1	119.32 (17)	H15B—C15—H15C	109.5
C1—C6—N1	121.92 (15)	O2—C16—O1	122.99 (17)
N1—C7—N2	125.29 (16)	O2—C16—C14	126.50 (18)
N1—C7—N3	120.91 (15)	O1—C16—C14	110.52 (16)
N2—C7—N3	113.72 (15)	O1—C17—C18	107.19 (18)
N3—C8—C14	109.22 (13)	O1—C17—H17A	110.3
N3—C8—C1	105.62 (13)	C18—C17—H17A	110.3
C14—C8—C1	117.33 (14)	O1—C17—H17B	110.3
N3—C8—H8	108.1	C18—C17—H17B	110.3
C14—C8—H8	108.1	H17A—C17—H17B	108.5
C1—C8—H8	108.1	C17—C18—H18A	109.5
N2—C9—C10	107.66 (17)	C17—C18—H18B	109.5
N2—C9—C11	111.32 (17)	H18A—C18—H18B	109.5
C10—C9—C11	112.96 (19)	C17—C18—H18C	109.5
N2—C9—H9	108.3	H18A—C18—H18C	109.5
C10—C9—H9	108.3	H18B—C18—H18C	109.5
C11—C9—H9	108.3	C7—N1—C6	116.54 (15)
C9—C10—H10A	109.5	C7—N2—C9	123.11 (15)
C9—C10—H10B	109.5	C7—N2—H2A	117.5 (13)
H10A—C10—H10B	109.5	C9—N2—H2A	118.4 (13)
C9—C10—H10C	109.5	C12—N3—C7	118.27 (14)
H10A—C10—H10C	109.5	C12—N3—C8	118.45 (14)
H10B—C10—H10C	109.5	C7—N3—C8	110.19 (13)
C9—C11—H11A	109.5	C12—N4—C13	125.35 (15)
C9—C11—H11B	109.5	C12—N4—H4A	114.4 (14)
H11A—C11—H11B	109.5	C13—N4—H4A	120.2 (14)
C9—C11—H11C	109.5	C16—O1—C17	116.80 (15)

### Hydrogen-bond geometry (Å, º)

**Table:**

*D*—H···*A*	*D*—H	H···*A*	*D*···*A*	*D*—H···*A*
N2—H2*A*···O2^i^	0.86 (1)	2.26 (1)	3.102 (2)	165 (2)
N4—H4*A*···S1^ii^	0.87 (1)	2.42 (1)	3.2804 (15)	172 (2)

Symmetry codes: (i) −*x*, −*y*+1, −*z*+2; (ii) −*x*+1, −*y*+1, −*z*+2.
